# A radiomics-based deep learning approach to predict progression free-survival after tyrosine kinase inhibitor therapy in non-small cell lung cancer

**DOI:** 10.1186/s40644-023-00522-5

**Published:** 2023-01-20

**Authors:** Chia-Feng Lu, Chien-Yi Liao, Heng-Sheng Chao, Hwa-Yen Chiu, Ting-Wei Wang, Yen Lee, Jyun-Ru Chen, Tsu-Hui Shiao, Yuh-Min Chen, Yu-Te Wu

**Affiliations:** 1 Department of Biomedical Imaging and Radiological Sciences, National Yang Ming Chiao Tung University, Taipei, Taiwan; 2 Department of Chest Medicine, Taipei Veteran General Hospital, Taipei, Taiwan; 3 Institute of Biophotonics, National Yang Ming Chiao Tung University, Taipei, Taiwan; 4 School of Medicine, National Yang Ming Chiao Tung University, Taipei, Taiwan; 5 Brain Research Center, National Yang Ming Chiao Tung University, Taipei, Taiwan

**Keywords:** Computer tomography imaging, EGFR TKI, Deep learning, Radiomics, Prognostic

## Abstract

**Background:**

The epidermal growth factor receptor (*EGFR*) tyrosine kinase inhibitors (TKIs) are a first-line therapy for non-small cell lung cancer (NSCLC) with *EGFR* mutations. Approximately half of the patients with *EGFR*-mutated NSCLC are treated with EGFR-TKIs and develop disease progression within 1 year. Therefore, the early prediction of tumor progression in patients who receive EGFR-TKIs can facilitate patient management and development of treatment strategies. We proposed a deep learning approach based on both quantitative computed tomography (CT) characteristics and clinical data to predict progression-free survival (PFS) in patients with advanced NSCLC after EGFR-TKI treatment.

**Methods:**

A total of 593 radiomic features were extracted from pretreatment chest CT images. The DeepSurv models for the progression risk stratification of EGFR-TKI treatment were proposed based on CT radiomic and clinical features from 270 stage IIIB-IV *EGFR*-mutant NSCLC patients. Time-dependent PFS predictions at 3, 12, 18, and 24 months and estimated personalized PFS curves were calculated using the DeepSurv models.

**Results:**

The model combining clinical and radiomic features demonstrated better prediction performance than the clinical model. The model achieving areas under the curve of 0.76, 0.77, 0.76, and 0.86 can predict PFS at 3, 12, 18, and 24 months, respectively. The personalized PFS curves showed significant differences (*p* < 0.003) between groups with good (PFS > median) and poor (PFS < median) tumor control.

**Conclusions:**

The DeepSurv models provided reliable multi-time-point PFS predictions for EGFR-TKI treatment. The personalized PFS curves can help make accurate and individualized predictions of tumor progression. The proposed deep learning approach holds promise for improving the pre-TKI personalized management of patients with *EGFR*-mutated NSCLC.

**Supplementary Information:**

The online version contains supplementary material available at 10.1186/s40644-023-00522-5.

## Introduction

Lung cancer is the most common malignant neoplastic disease worldwide and is categorized into small cell lung cancer and non-small cell lung cancer (NSCLC), causing nearly 2 million deaths globally each year [1]. Most NSCLC patients develop relapse of the disease after surgery or are even diagnosed as medically inoperable and therefore have to receive systemic therapies [2]. Patients with advanced or metastatic NSCLC have to receive systemic therapies for tumor control. The development of targeted therapies over the last two decades has contributed considerably to the management of NSCLC patients. Epidermal growth factor receptor (*EGFR*) mutations, mainly exon 19 deletion and exon 21 L858R mutations, are the most commonly detected oncogenic drivers in approximately 20%–50% of stage IV NSCLC patients. Previous studies have indicated that nearly half of NSCLC patients in Asia have *EGFR* mutations [3, 4]. *EGFR* tyrosine kinase inhibitors (TKIs) have been demonstrated to suppress the growth of NSCLC with *EGFR* mutations [5]. EGFR mutations are more prevalent in Asian NSCLC patients [6]. Accordingly, the application of TKI therapy in NSCLC has received a great deal of attention, especially in East Asia [7].

The common first-line EGFR-TKI therapy for NSCLC includes gefitinib, erlotinib, and afatinib. In phase III clinical trials, patients receiving these medications have achieved overall response rates of 56% to 74%, progression-free survival (PFS) of 9.7 to 11.1 months, and overall survival of 22.9 to 28.2 months [8–10]. However, resistance to EGFR-TKI in patients with NSCLC is frequently observed within 1 year after treatment [11, 12]. Therefore, early identification of patients with a high probability of tumor progression after EGFR-TKI therapy can facilitate the development of appropriate treatment strategies and is therefore crucial for the management of advanced NSCLC. Additionally, intra-tumor heterogeneity among the postulated molecular mechanisms have been found to be associated with resistance to EGFR-TKI therapy [13]. However, some studies have observed that clinical prognostic factors for evaluating EGFR-TKI resistance only possess a limited predictive effect because of the interplay of molecular mechanisms in NSCLC [14, 15]. In recent years, quantitative radiomics analysis of medical images has been considered as a promising non-invasive diagnostic method for the study of primary or metastatic lung cancer [16–18]. The proposed Image Biomarker Standardization Initiative (IBSI) improves the reliability of radiomics analysis and further accentuates its clinical applications based on image quantification [19]. Moreover, radiomic features extracted from computed tomography (CT) images are suggested to evaluate the heterogeneity of lung lesions [20, 21]. These features can also be applied as independent predictors to complement clinical information.

In this study, we proposed a deep learning-based approach to assess the personalized probability of tumor progression in patients who had advanced NSCLC with *EGFR* mutations treated with EGFR-TKIs. The proposed models combining chest CT radiomic and clinical features provided a reliable prediction of PFS. We hypothesized that imaging features extracted from pretreatment chest CT could improve the prediction of tumor progression after EGFR-TKI treatment in NSCLC patients.

## Materials and methods

### Study design

**Fig. 1:**
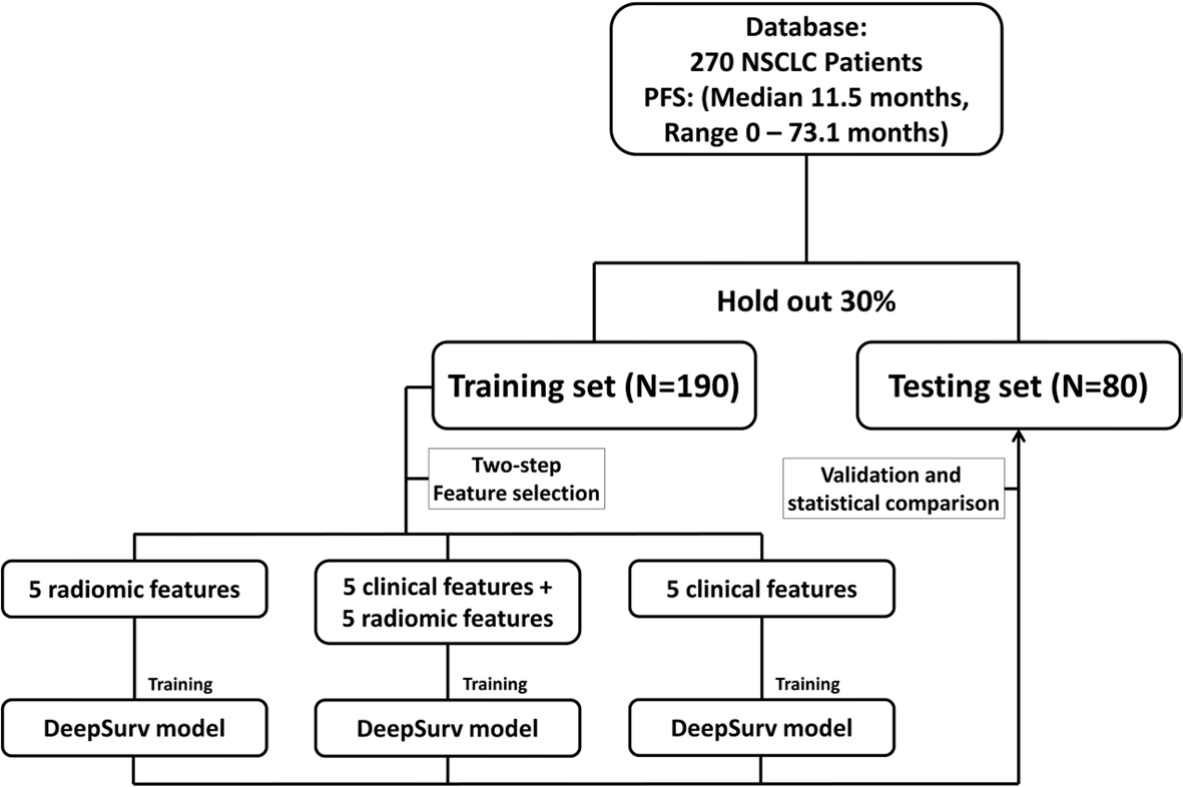
Flowchart for development and validation of DeepSurv model

The Institutional Review Board of Taipei Veterans General Hospital approved this retrospective study (2021–09-009BCF) and waived the requirement of acquiring informed consent from patients. The design of this study is shown in Fig. 1, which includes the inclusion of patients, the collection of clinical data and standardized contrast CT imaging features, creation of independent training and testing datasets, selection of key features for predicting PFS in the training dataset, development of deep learning models based on clinical features alone or in combination with CT radiomics, and assessment of the effectiveness of the PFS prediction after TKI treatment in the testing data set. The estimated personalized PFS curves of the model were applied to predict the progression risk period and the short-term (3 months), medium-term (12 months), and long-term (18 and 24 months) progression status. This study was performed in accordance with the Declaration of Helsinki [22].

### Patient cohort and image data

This study retrospectively included 270 *EGFR*-mutated NSCLC patients treated with EGFR-TKIs from 2017 to 2020. The patient data were collected in accordance with the following inclusion criteria: (1) identification of NSCLC with a stage greater than IIIB according to the American Joint Committee on Cancer (AJCC) staging system, edition 8 [23]; (2) evidence from histological examinations of pathology samples from surgical specimens or tissue biopsies; (3) receipt of first-line EGFR-TKI treatment without surgery, chemotherapy, and radiotherapy for NSCLC in accordance with the National Comprehensive Cancer Network (NCCN) treatment guidelines [24]; (4) adequate quality contrast chest CT examination data and clinical information; and (5) patient without other neoplastic diseases.

The quality assessment of CT scans and delineation of the regions of interest (ROIs) was performed by a multidisciplinary team of experienced radiologists and certified pulmonologists. The soft tissue window (width: 350, level: 50) and lung window (width: 1500, level: − 600) on CT images were applied for ROI delineation. The soft tissue window was used to distinguish between tumors, collapsed lungs, and fluid components, such as pleural and pericardial effusions, and the lung window was applied to determine the border of tumors.

### Radiomics analysis and feature selection

The acquired CT images were subjected to numerous preprocessing steps before radiomics analysis. First, the resolution of the CT images was adjusted to the identical dimension with a pixel size of 1 × 1 × 1 mm^3^. Second, the intensities of the CT images were converted into standardized ranges (Z-score transformation) based on the mean and standard deviation of image data. Finally, low-pass (L) and high-pass (H) dimensional wavelet filtering were applied to the three axes of CT images, producing eight image sets: LLL, LLH, LHL, LHH, HLL, HLH, HHL, and HHH wavelet filtered images.

Radiomic features, including histogram, geometry, and texture features (gray level co-occurrence matrix, GLCM; gray level run length matrix, GLRLM; and local binary pattern, LBP) [25, 26], were extracted from all image data sets (eight wavelet decomposed and original CT images). In the feature extraction process, the feature aggregation of GLCM and GLRLM values was performed by averaging over 3D directional matrices according to IBSI guideline to achieve optimal rotational invariance [19]; slice-by-slice computation of the LBP features was followed by histogram analysis of the LBP matrix for all CT slices. A total of 593 radiomic features were generated for each ROI. All of the ROI delineation, image preprocessing, and subsequent radiomics analysis were performed using the previously published Multimodal Radiomics Platform (available online: http://cflu.lab.nycu.edu.tw/MRP_MLinglioma.html, accessed on 6 Sept 2022) [18, 27] in compliance with the IBSI on the MATLAB R2022a environment [19]. The formulae for radiomics analysis are listed in Table S1.

To identify key radiomic and clinical features for predicting TKI outcomes, a two-step feature selection was applied to the training data set (70% of cases). The initial statistical method selections, including using univariate Cox proportional regression for radiomic features and the chi-squared test for clinical features, were followed by the implementation of a sequential forward selection (SFS) algorithm [28]. Moreover, to maintain the validity of the deep learning model (i.e., a sufficient number of input features), we applied the selection criterion of *p* < 0.1 in the first step (the Cox and chi-squared methods). Then, the performance of the proposed PFS prediction models was evaluated using a testing data set (the remaining 30% of cases).

### Prediction models

The DeepSurv model, a multilayer perceptron based on the Cox proportional hazards (CPH), was applied to estimate tumor progression after TKI treatment [29]. Conventional CPH model contains a log-linear regression of relative hazard function that links covariates to the patient survival. The DeepSurv model substitutes the CPH log-linear regression with a multi-layer perceptron to estimate the nonlinear properties of the hazard function, and thus has the potential to achieve superior performance in survival prediction. DeepSurv is a configurable feed-forward neural network and the input to the network is the baseline predictors. The network propagates the input data through several hidden layers with specified weights. The hidden layers include batch normalization, nonlinear rectified linear unit (ReLU) activation, fully connected, and dropout layers. The final layer is a single node that conducts a linear combination to generate the final output. The hyper-parameters influence the performance of DeepSurv model with regard to the training time, model convergence speed, and prediction accuracy. Accordingly, the optimization of hyper-parameters is essential for model training.

In this study, the hyper-parameters of network (including number of hidden layers, number of nodes in each layer, initial learning rate, learning rate decay, and dropout rate) were determined using the grid search method [30]. The setup of hyper-parameters was determined based on the prediction performance and training time cost (Table S2). Finally, the proposed DeepSurv perceptron consisted of an input layer (the number of nodes was equal to the number of selected features), hidden layers (including batch normalization, ReLU activation, 32-node fully connected, and dropout layers), and an output layer. Moreover, an Adam optimizer with a dropout rate of 40%, an initial learning rate of 0.01, a learning rate decay of 0.01, and L2 regularization was performed in the training process. The loss function of DeepSurv models was defined as the average negative log partial likelihood proposed in a previous study [29]. The architecture of proposed DeepSurv model is shown in Figure S1.

Three DeepSurv models were developed using the following information: (1) clinical features (e.g., AJCC TNM stage, smoking status, and the histopathology of NSCLC); (2) radiomic features; and (3) a combination of clinical and radiomic features. The DeepSurv models are used to estimate personalized PFS curves for each case based on the corresponding logarithmic hazard function. A log-rank test was performed to assess the statistical difference in the average of personalized PFS curves between the good control (PFS > 11.5 months) and poor control (PFS < 11.5 months) groups.

The DeepSurv models were further applied to predict the progression status at individual follow-up time points (e.g., 3, 12, 18, and 24 months). The predictive efficacy of the DeepSurv models in predicting progression status was evaluated using time-dependent receiver operating characteristic (ROC) curves, area under the ROC curve (AUC), index of concordance (C-index), sensitivity, and specificity. A bootstrap random sampling method was applied to the testing data set to statistically compare the prediction performance of the clinical/radiomic and combined (clinical and radiomic features) DeepSurv models [31]. A paired *t* test was used to compare the difference in the AUC between models. The feature selection and subsequent DeepSurv model training were performed on R DeepSurv package (available online: https://rdrr.io/cran/survivalmodels/src/R/deepsurv.R, accessed on 6 Sept 2022).

The optimal thresholds for time-dependent ROC curves at each selected time point were applied to intuitively represent personalized progression risks. A Weibull probability distribution function was applied for curve fitting through the four time-dependent thresholds to construct a reference risk curve [32]. The area between the reference risk curve and the personalized PFS curve was used to assess the risk of tumor progression. If the value of this area during the observed period was negative, it indicated that the part of the personalized PFS curve was lower than the reference risk curve and represented a high risk of progression. A schematic representation of the risk-of-progression period is shown in Figure S2.

## Results

### Clinical characteristics of recruited patients

In the present study, the median PFS of the recruited patients with NSCLC after TKI treatment was 11.5 months. Over 74% of patients were nonsmokers. The majority of patients had stage IV adenocarcinoma (97.4%) and exhibited EGFR exon 19 deletions (43.3%) and exon 21 L858R substitutions (49.3%). Over half of the patients showed no adverse effects after TKI treatment. Table 1 summarizes the clinical characteristics of the 270 recruited NSCLC patients. No significant differences in clinical characteristics were identified between the training and test sets (Table S3). The results of clinical laboratory tests are listed in Table S4.

**Table 1:** Characteristics of 270 recruited NSCLC patients

Characteristics	Value
**Age, median(IQR)**	67.5 (60–75)
**Gender**	
Female, N(%)	158 (58.5)
**Smoking status**	
Smoker, N(%)	69 (25.6)
**ECOG PS score**	
0, N(%)	98 (36.3)
1, N(%)	139 (51.5)
2, N(%)	22 (8.1)
> 2, N(%)	11 (4.1)
**Histology of NSCLC**	
Adenocarcinoma, N(%)	263 (97.4)
Squamous cell carcinoma, N(%)	4 (1.5)
Others, N(%)	3 (1.1)
**Clinical T stage**	
1, N(%)	35 (13.0)
2, N(%)	78 (28.9)
3, N(%)	44 (16.3)
4, N(%)	106 (39.2)
Not available, N(%)	7 (2.6)
**Clinical N stage**	
0, N(%)	69 (25.6)
1, N(%)	20 (7.4)
2, N(%)	75 (27.8)
3, N(%)	104 (38.5)
Not available, N(%)	2 (0.7)
**Clinical M stage**	
0, N(%)	10 (3.7)
1a, N(%)	82 (30.4)
1b, N(%)	41 (15.2)
1c, N(%)	137 (50.7)
**Clinical stage**	
Stage III, N(%)	23 (8.5)
Stage IVA, N(%)	110 (40.7)
Stage IVB, N(%)	137 (50.8)
**EGFR mutation status**	
Exon 19 deletion, N(%)	117 (43.3)
Exon 21 L858R substitution, N(%)	133 (49.3)
Others, N(%)	20 (7.4)
**TKI**	
Gefitinib, N(%)	46 (17.0)
Erlotinib, N(%)	85 (31.5)
Afatinib, N(%)	139 (51.5)
**Adverse drug reaction to EGFR-TKI**	
Yes, N(%)	130 (48.1)
**Progression free survival, median(months)**	11.5 (4.9–17.9)
**Total protein**	
High, N(%)	87 (32.2)
Low, N(%)	6 (2.2)
Not available, N(%)	177 (65.6)
**Mean corpuscular volume**	
High, N(%)	85 (31.5)
Normal, N(%)	81 (30.0)
Low, N(%)	5 (1.9)
Not available, N(%)	99 (37.6)
**White blood cells count**	
High, N(%)	54 (20.0)
Normal, N(%)	185 (68.5)
Low, N(%)	7 (2.6)
Not available, N(%)	24 (8.9)

Definitions: total proteins: high: > 6 g/dl, low: < 6 g/dl; mean corpuscular volume: high > 100 fl, normal:80-100 fl, low < 80 fl; white blood cells count: high: > 12,000/cumm, normal 4000–12,000/cumm, low: < 4000/cumm

*Abbreviations*: *ECOG* Eastern Cooperative Oncology Group, *EGFR* Epidermal growth factor receptor, *NSCLC* Non-small cell lung cancer, *PS* Performance status, *TKI* Tyrosine kinase inhibitor

### Selected features for PFS prediction

We ultimately selected 10 features, including 5 clinical and 5 radiomic features, through the two-step feature selection process. The key clinical features for predicting tumor progression included regional lymph node metastasis, distant metastasis of the tumor, NSCLC histology, total protein, and mean corpuscular volume. Selected radiomic features included textural features that described local homogeneity and one geometric feature that measured the compactness of tumor shape compared to a sphere. Four of the five selected radiomic features were GLCM features—GLCM inverse difference moment normalized (IDMN) based on LHL, HLL, HHL, and HHH wavelets—and the remaining one is a geometry feature—compactness. The details of the selected features are listed in Table S5.

### Performance of DeepSurv prediction models

**Fig. 2:**
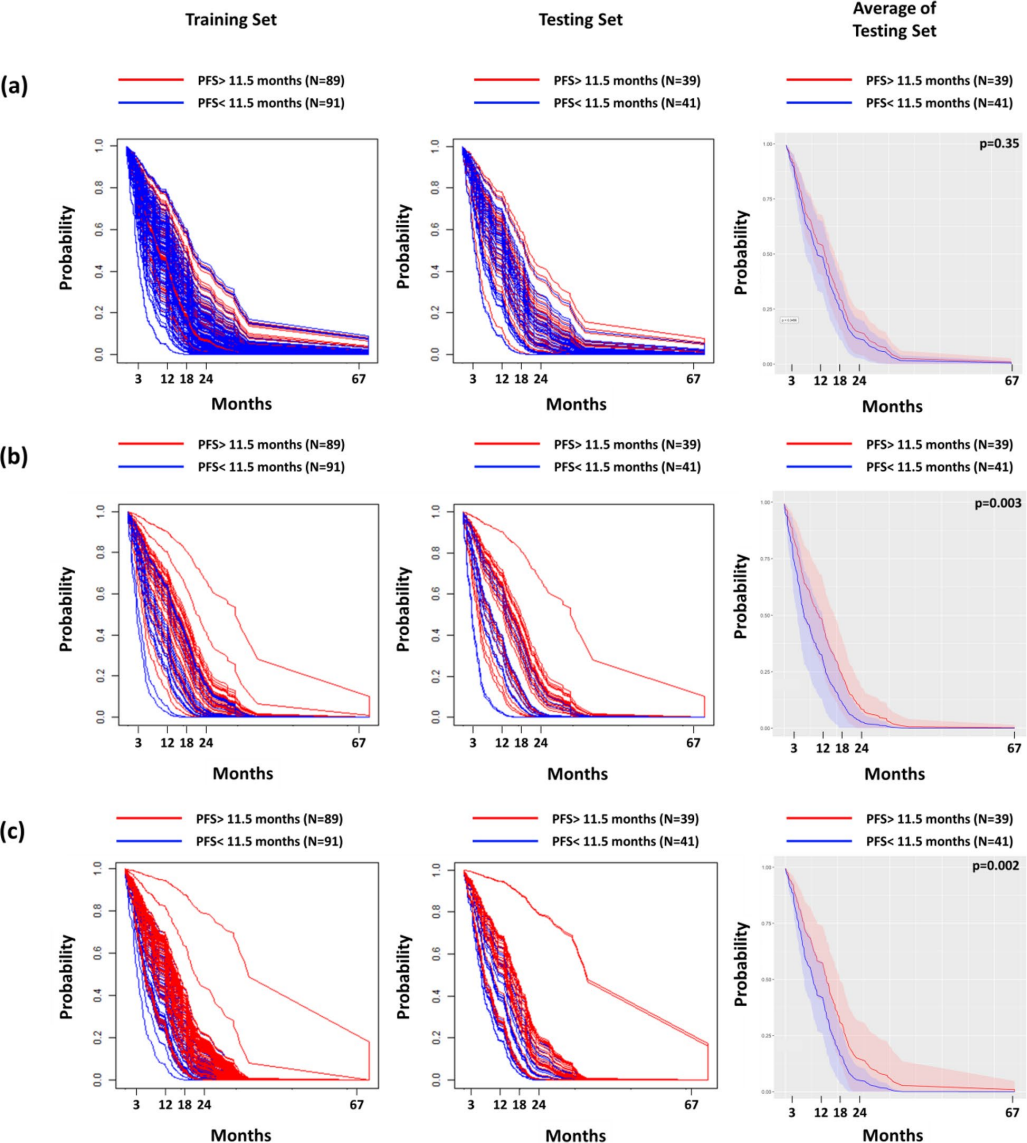
Distribution of personalized PFS curves predicted by the DeepSurv models. The estimated personalized PFS curves of patients in training set, testing set, and average of personalized PFS curves in testing set based on (**a**) radiomic, (**b**) clinical, and (**c**) combined model, respectively. The red curves in the figure represented the patients with PFS better than median PFS, and blue curves indicated the patients with PFS poorer than median PFS

The patients were divided into good control (PFS > 11.5 months) and poor control (PFS < 11.5 months) groups based on the median PFS, and we sought to evaluate the prediction efficacy of the DeepSurv prediction models. Figure 2 displays the personalized PFS curves estimated using the DeepSurv models based on the radiomic, clinical, and combined (clinical and radiomic features) datasets, respectively. Our results demonstrated that personalized survival curves generated by clinical and combined DeepSurv models differed significantly between the two tumor control groups (*p* < 0.002), which indicated that both models provided reliable predictions in differentiating tumor responses to TKI treatment. However, average PFS curves estimated by the model solely based on radiomic features were not significantly different (*p* = 0.35) between the good and poor control groups.

**Fig. 3:**
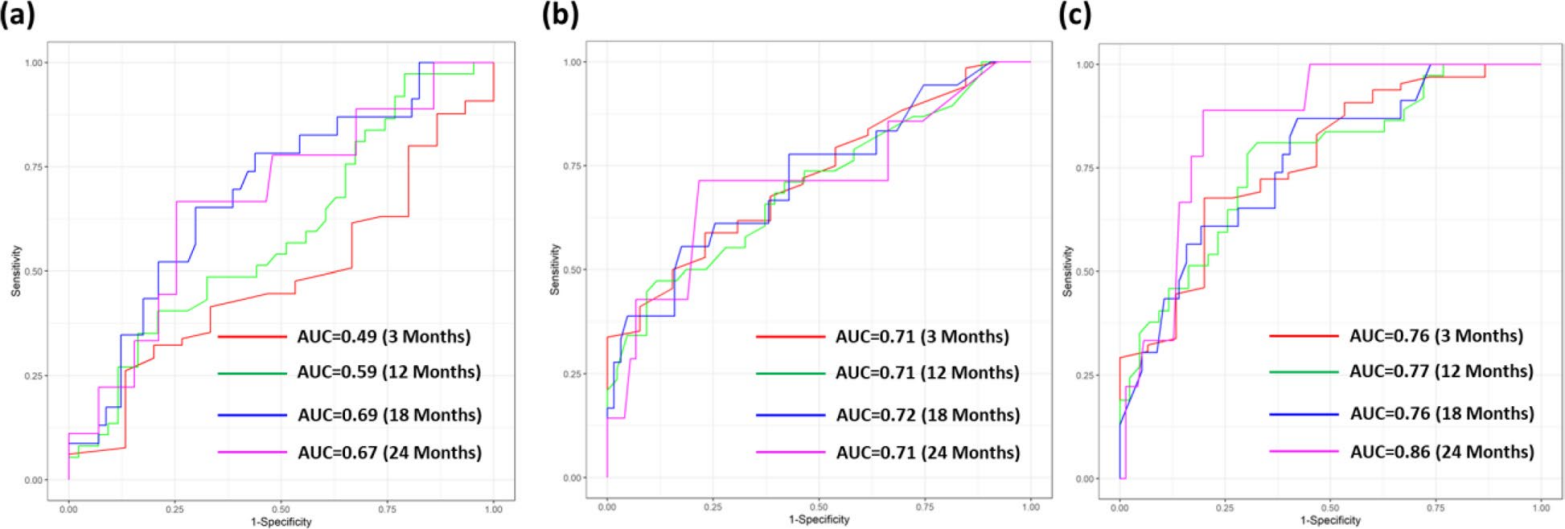
Results of time-dependent prediction of PFS after TKI treatment. The time-dependent ROC curves of DeepSurv models for predicting PFS after TKI treatment based on (**a**) radiomic, (**b**) clinical, and (**c**) combined model, respectively

We estimated the prediction performance at four follow-up time points (3, 12, 18, and 24 months) by using the testing data set. The time-dependent ROC curves for each model are shown in Fig. 3. The radiomic feature-based model produced AUCs between 0.49 and 0.69 with C-index of 0.57. The clinical feature-based model produced AUCs between 0.71 and 0.72 with a C-index of 0.63. The combined model had an AUC range of 0.76 to 0.86 with a C-index of 0.66. Table 2 lists the comprehensive performance and statistical comparisons between radiomic/clinical and combined models. Overall, the combined model significantly outperformed the radiomic and clinical models in terms of efficacy (AUC, sensitivity, and specificity) in predicting progression risk at each selected time point.

**Table 2:** Statistical comparisons between developed prediction models based on test dataset

Model performance	Radiomic (C-index = 0.57)	Clinical (C-index = 0.63)	Combined (C-index = 0.66)	*p*-values	
				Radiomic vs. Combined	Clinical vs. Combined
3 months					
Original AUC	0.49	0.71	0.76		
AUC	0.52 ± 0.05	0.70 ± 0.06	0.75 ± 0.06	< 0.001^a^	< 0.001^a^
Sensitivity	0.48 ± 0.10	0.66 ± 0.10	0.68 ± 0.05	< 0.001^a^	0.03^a^
Specificity	0.64 ± 0.09	0.69 ± 0.06	0.73 ± 0.13	< 0.001^a^	< 0.001
12 months					
Original AUC	0.59	0.71	0.77		
AUC	0.60 ± 0.11	0.70 ± 0.06	0.78 ± 0.05	< 0.001^a^	< 0.001^a^
Sensitivity	0.51 ± 0.04	0.67 ± 0.11	0.69 ± 0.06	< 0.001^a^	0.02^a^
Specificity	0.60 ± 0.08	0.62 ± 0.07	0.70 ± 0.11	< 0.001^a^	< 0.001^a^
18 months					
Original AUC	0.69	0.72	0.76		
AUC	0.70 ± 0.05	0.71 ± 0.08	0.78 ± 0.05	< 0.001^a^	< 0.001^a^
Sensitivity	0.60 ± 0.10	0.67 ± 1.12	0.65 ± 0.06	< 0.001^a^	0.07
Specificity	0.67 ± 0.03	0.62 ± 0.06	0.80 ± 0.14	< 0.001^a^	< 0.001^a^
24 months					
Original AUC	0.67	0.71	0.86		
AUC	0.70 ± 0.10	0.69 ± 0.13	0.85 ± 0.05	< 0.001^a^	< 0.001^a^
Sensitivity	0.71 ± 0.08	0.71 ± 0.13	0.89 ± 0.06	< 0.001^a^	< 0.001^a^
Specificity	0.75 ± 0.13	0.78 ± 0.06	0.80 ± 0.14	< 0.001^a^	0.03^a^

^a^Significant difference based on the paired t-test

**Fig. 4:**
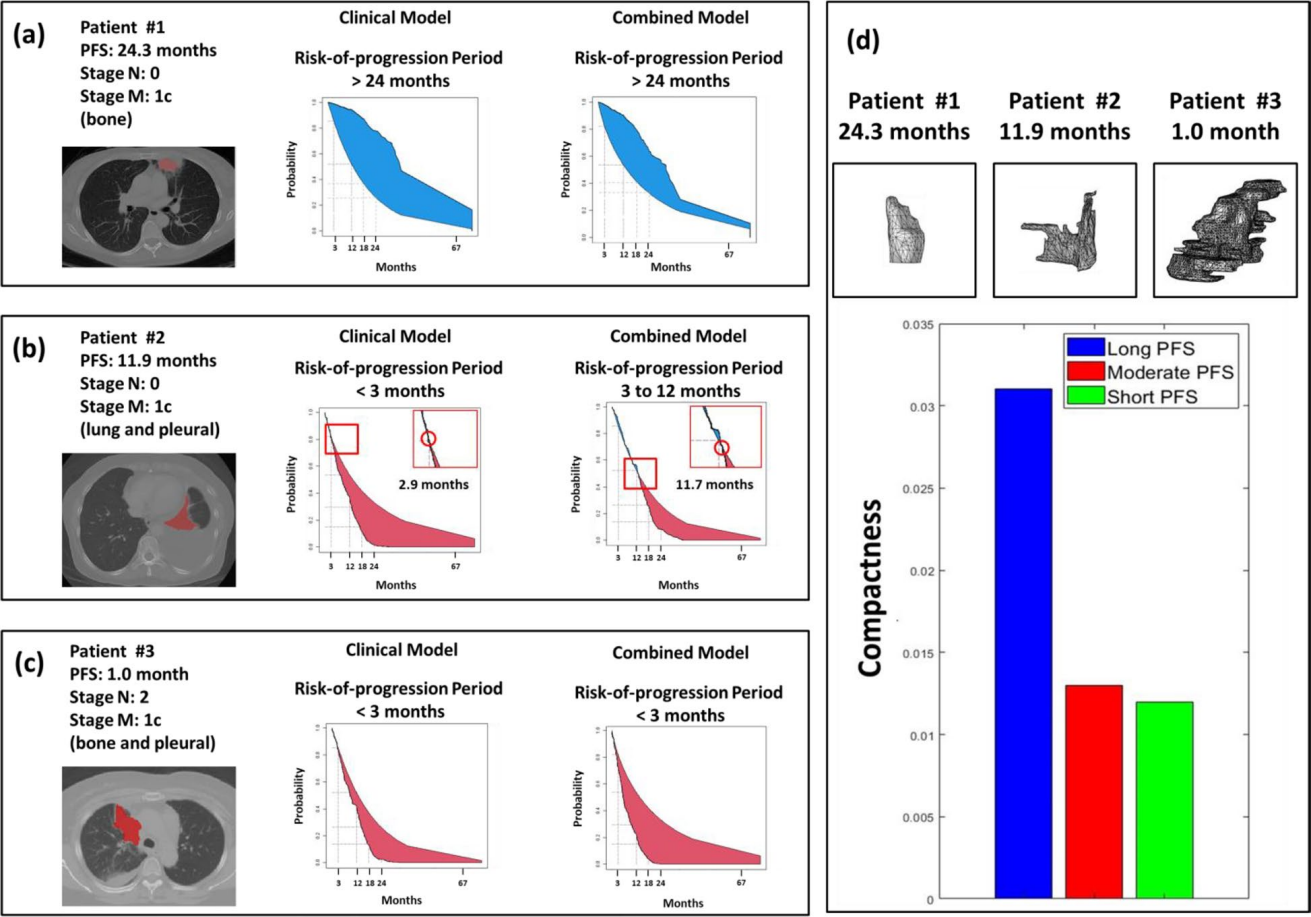
Representative cases for the predictions of PFS based on different data set. Figure shows CT images and the DeepSurv risk-of-progression period of (**a**) a patient with long (24.3 months) PFS, (**b**) a patient with moderate (11.9 months) PFS, and (**c**) a patient with short (1.0 month) PFS. The comparison of the selected geometric feature with the PFS of representative cases is presented in (**d**)

Figure 4 illustrates the prediction of the risk-of-progression period for representative cases with long (24.3 months), moderate (11.9 months), and short PFS (1.0 month) time. For patients with a long PFS (without any regional lymph node metastasis or bone metastases, Fig. 4a), both clinical and combined models generated personalized PFS curves that were higher than the reference risk curve. This indicated the models accurately predicted a risk-of-progression period of longer than 24 months. For patients with a moderate PFS (without any regional lymph node metastasis but having lung and pleural metastases, Fig. 4b), only the combined model identified an intersection between the personalized PFS curve and the reference risk curve during the period of 3 to 12 months. This indicated that two models accurately predicted a risk-of-progression period between 3 and 12 months. As for patients with a short PFS (having regional lymph node metastases and bone and pleural metastases, Fig. 4c), both clinical and combined models estimated personalized PFS curves that were lower than the reference risk curves, indicating that the accurate prediction of a risk-of-progression period was less than 3 months. Patients with long PFS had a higher value of compactness (one of the selected radiomic features) reflecting a rounder-shaped lesion than those with moderate or short PFS (Fig. 4d). The results suggested that the combined models provided reliable estimates of the risk-of-progression period for patients with NSCLC after EGFR-TKI therapy.

## Discussion

First-generation EGFR-TKIs (gefitinib and erlotinib) and the second-generation EGFR-TKI( afatinib) have been used as the first-line treatment of NSCLC in the last decade [33, 34]. Patients with *EGFR*-mutant NSCLC who were treated with EGFR-TKIs had an improved PFS compared with those treated with standard chemotherapy [35]. The most common reason for discontinuing EGFR-TKI therapy is tumor progression, and therefore, personalized prediction of EGFR-TKI resistance is notable [36]. Hence, a reliable prediction could prevent potential adverse drug reactions and facilitate the early implementation of necessary treatments. In patients with NSCLC, contrast-enhanced chest CT remains the standard imaging test for the initial diagnosis of NSCLC. Nevertheless, according to our survey, CT images without contrast enhancement constitute the majority of the publicly accessible NSCLC imaging databases. Furthermore, to enrich the imaging database of NSCLC, a prognostic model based on contrast-enhanced CT images should be proposed that considers the possibility of combining multimodality CT into the data set.

Studies have revealed the potential of radiomic features extracted from CT images to predict outcomes of TKI therapy in patients with advanced NSCLC [37, 38]. The multivariate CPH models, the most extensively used survival analysis approach, have been applied in several studies. However, the calculation of linear covariance between variables using the CPH model does not provide a reliable assessment of therapeutic outcomes because the covariation between prognostic factors is mostly nonlinear. Moreover, this limitation becomes more evident when high-throughput radiomic features are further exploited as prognostic factors in the regression model. Therefore, we applied the DeepSurv model to predict tumor progression in NSCLC patients. The DeepSurv model that features a multilayer neural network provides a reliable nonlinear regression of covariates between prognostic factors [29]. Furthermore, estimated personalized PFS curves from the DeepSurv model provide an intuitive approach for prognostic evaluation. Estimated risk-of-progression periods are allowed for the prediction of TKIs resistance in NSCLC patients for personalizing treatment strategies and management.

In a previous study, clinical-based CPH models with a C-index of 0.62 to 0.63 were proposed to predict PFS after EGFR-TKI treatment in NSCLC patients. A CPH model based on CT radiomics has been further used for time-dependent PFS prediction. The models achieved AUCs ranging from 0.70 to 0.82 in predicting PFS at 10 and 12 months. This indicated different data sets could lead to bias in the prediction performance of the model [38]. Our proposed combined model exhibited a more accurate prediction performance than the clinical and radiomic models and achieved a C-index of 0.66. Moreover, the model had reliable efficacy in predicting PFS at 3, 12, 18, and 24 months (achieving AUCs of 0.75–0.86), and its high prediction performance could be attributed to two reasons. First, the radiomics process in the present study was conducted in accordance with the IBSI guideline [19]. Standardized image quantification enhanced the stability of radiomic features and the reliability of prediction models. Second, we applied the DeepSurv model to simulate the nonlinear interactions between predictors. This may facilitate the adaptation of models to changes in the risk of tumor progression at different time points.

In the two-step feature selection process, the histology of NSCLC and the AJCC pathological N and M stages were identified as the key clinical factors for predicting PFS after EGFR-TKI treatment. The presence of squamous NSCLC and lymph node metastases are known prognostic factors for advanced lung cancer [39, 40]. We further categorized the metastatic states as M0, M1a, M1b, and M1c staging based on the number of occurrences location [41]. Our results indicated that patients with multiple distant metastases had a poor prognosis. Moreover, we considered two commonly used laboratory features, namely total protein and red blood cell mean corpuscular volume, in the analysis. Patients with low total protein and high mean corpuscular volume are associated with poor PFS. A low total protein level may reflect patient exhaustion, which may cause patients to have severe constitutional symptoms and the inability to withstand intensive treatment [42]. Increasing values of red blood cell mean corpuscular volume may indicate a deficiency of folate, resulting in abnormal methylation, synthesis, replication, and repair of DNA [43, 44]. However, because not all patients received hematology tests, the association of these features with TKI treatment outcomes requires further investigation. Smoking, a known independent prognostic factor for NSCLC, was not considered for the following reasons. First, the feature data set may contain indicators that are highly correlated with smoking; therefore, smoking was given less weightage in the SFS algorithm. Second, only approximately 25% of the patients in this study were smokers, which could have affected prediction accuracy due to data imbalance. Third, smokers were related to a high incidence of non-EGFR-mutant lung cancers [45], implying that this factor had a confounding effect. Hence, tumor stage and laboratory data may be considered to assess the efficacy of TKI therapy in NSCLC patients.

Even though the radiomic model itself was not sufficient to accurately predict the PFS, our results demonstrated that the synergetic effect of combined model (including both radiomic and clinical features) showed significant enhancement of prediction performance. Compactness and IDMN were the selected radiomic features for PFS prediction. The results revealed that patients with poor PFS had reduced values of compactness in their CT testing. In addition, low compactness indicates that the tumor exhibits a more asymmetric geometry relative to a spherical tumor and has been reported to be associated with a highly aggressive form of tumor [46]. This finding implied that the high local aggressiveness of NSCLC was one of the main causes of EGFR-TKI resistance. In addition, patients with poor PFS exhibited high values of IDMN on CT scans. High IDMN values indicate that the voxel intensity of the image is locally similar. *EGFR* mutated NSCLC is recognized to be highly angiogenic and venous aggressive [47] and is linked to a low IDMN value on contrast CT images. Therefore, NSCLC patients with low IDMN values on CT images can be expected to have a high level of EGFR mutations and a good EGFR-TKI response.

In this study, the application of DeepSurv model was suggested to evaluate the risk-of-progression period of NSCLC patients. Estimated personalized PFS curves describe the probability of tumor progression after EGFR-TKI treatment. As tumor progression may occur at different times during follow-up, time-dependent ROC curves can be used to assess the progression status at critical follow-up time points. Figure 4 indicates that the clinical model provided a reliable PFS prediction for patients with good and poor tumor control. This implied that the prognostic effect of different stages of conventional tumor staging was significant. The clinical prediction model performed poorly because patients with moderate tumor control frequently presented at similar clinical stages. The DeepSurv model incorporating radiomic features provided information on tumor heterogeneity. The combined model also incorporated the tumor heterogeneity data from radiomics, which allowed the model to more effectively differentiate the prognosis between patients with similar tumor stages.

Several limitations and further considerations of this study are discussed as follows. First, the CT images and therapeutic information of patients in this study were acquired from a single institution. The proposed models should be validated with an external validation data set from multiple centers in future research. Second, the tumor segmentation in this study was performed manually by a multidisciplinary team of experienced pulmonologist and radiologists based on different CT windows. The development of an automated CT image segmentation method could reduce the time required for manual segmentation and improve the reproducibility and robustness of radiomic features. Finally, clinical laboratory information of all patients was not available due to the retrospective nature of the study. Future studies are expected to prospectively collect the proposed key clinical aspects of data.

## Conclusions

The information on the staging, histology, and blood analysis results of NSCLCs patients could be used to provide a reliable prediction of possible tumor progression after EGFR-TKI treatment. The additional inclusion of quantitative CT characteristics describing tumor compactness and local homogeneity further improved the predictive performance of the models. The risk-of-progression period based on the DeepSurv model can provide personalized predictions of therapeutic outcomes after EGFR-TKI treatment in a more intuitive manner and may help personalize treatment strategies for advanced NSCLC patients who have received EGFR-TKI treatment.

## Supplementary Information

**Additional file 1: Table S1.** The formulae for the calculation of primary radiomic features. **Table S2.** Grid search results of DeepSurv hyper-parameters. **Table S3.** Comparisons of clinical characteristics between training and test sets. **Table S4.** Characteristics of clinical laboratory test. **Table S5.** Identified features for the model training in each DeepSurv model. **Figure S1.** The architecture of applied DeepSurv model. **Figure S2.** Schematic diagram of predictive risk-of-progression period in DeepSurv model.

## Data Availability

The raw data cannot be made publicly available for ethical and legal reasons. However, researchers can submit inquiries for analyzed data to the corresponding authors upon reasonable request.

## Abbreviations

NSCLC: Non-small cell lung cancer
*EGFR*: Epidermal growth factor receptor
TKIs: Tyrosine kinase inhibitors
PFS: Progression-free survival
IBSI: Image Biomarker Standardization Initiative
CT: Computed tomography
AJCC: American Joint Committee on Cancer
NCCN: National Comprehensive Cancer Network
ROIs: Regions of interest
GLCM: Gray level co-occurrence matrix
GLRLM: Gray level run length matrix,
LBP: Local binary pattern
SFS: Sequential forward selection
CPH: Cox proportional hazards
ROC: Receiver operating characteristic
AUC: Area under the curve
C-index: Index of concordance
IDMN: Inverse difference moment normalized
