# Supplementary material for: A radiomics-based deep learning approach to predict progression free-survival after tyrosine kinase inhibitor therapy in non-small cell lung cancer
Source: Cancer Imaging. 2023 Jan 20;23:9. doi: 10.1186/s40644-023-00522-5 (PMC9854198; doi:10.1186/s40644-023-00522-5)
Supplement: Supplementary file 1 — Additional file 1: Table S1. The formulae for the calculation of primary radiomic features. Table S2. Grid search results of DeepSurv hyper-parameters. Table S3. Comparisons of clinical characteristics between training and test sets. Table S4. Characteristics of clinical laboratory test. Table S5. Identified features for the model training in each DeepSurv model. Figure S1. The architecture of applied DeepSurv model. Figure S2. Schematic diagram of predictive risk-of-progression period in DeepSurv model. [file 40644_2023_522_MOESM1_ESM.docx]

**SUPPLEMENTAL MATERIALS**

**for**

**Radiomics-Based Deep Learning Approach to Predict Progression Free Survival after Tyrosine Kinase Inhibitor Therapy in Non-small Cell Lung Cancer**

**by**

Chia-Feng Lu^#^、Chien-Yi Liao^#^、Heng-Sheng Chao、Hwa-Yen Chiu、Ting-Wei Wang、Yen Lee Jyun-Ru Chen、Tsu-Hui Shiao、Yuh-Min Chen^*^、Yu-Te Wu^*^

**Table S1** The formulae for the calculation of primary radiomic features.

| **Intensity-based features (first order statistics)**  **X** denotes the intensity vector with *N* voxels of the tumor ROIs; $\bar{X},$ the mean of **X**; **P**, the first-order histogram with *N_l_* discrete intensity levels. | | | | | |
| --- | --- | --- | --- | --- | --- |
| **Feature** | **Formula** | | **Feature** | | **Formula** |
| 1. Energy | $\sum_{i=1}^{N} \mathbf{X}{(i)}^{2}$ | | 2. Entropy | | $\sum_{i=1}^{N_{l}} \mathbf{P}(i){log}_{2}\mathbf{P}(i)$ |
| 3. Kurtosis | $\frac{\frac{1}{N}\sum_{i=1}^{N} \left( \mathbf{X}\left( i \right)-\bar{X} \right)^{4}}{\left( \sqrt{\frac{1}{N}\sum_{i=1}^{N} \left( \mathbf{X}\left( i \right)-\bar{X} \right)^{2}} \right)^{2}}-3$ | | 4. Maximum | | $max(\mathbf{X)}$ |
| 5. Mean | $\frac{1}{N}\sum_{i=1}^{N} \mathbf{X}(i)$ | | 6. Mean absolute deviation | | $\frac{1}{N}\sum_{i=1}^{N} abs(\mathbf{X}\left( i \right)-\bar{X})$ |
| 7. Median | $median(\mathbf{X})$ | | 8. First quartile | | Value that splits off the lowest 25% of data from the highest 75% |
| 9. Third quartile | Value that splits off the highest 25% of data from the lowest 75% | | 10. Minimum | | $min(\mathbf{X})$ |
| 11. Range | $\max\left( \mathbf{X} \right)-min(\mathbf{X})$ | | 12. Root mean square (RMS) | | $\sqrt{\frac{\sum_{i=1}^{N} \mathbf{X}{(i)}^{2}}{N}}$ |
| 13. Skewness | $\frac{\frac{1}{N}\sum_{i=1}^{N} \left( \mathbf{X}\left( i \right)-\bar{X} \right)^{3}}{\left( \sqrt{\frac{1}{N}\sum_{i=1}^{N} \left( \mathbf{X}\left( i \right)-\bar{X} \right)^{2}} \right)^{3}}$ | | 14. Standard deviation | | $\sqrt{\frac{1}{N}\sum_{i=1}^{N} \left( \mathbf{X}\left( i \right)-\bar{X} \right)^{2}}$ |
| 15. Uniformity | $\sum_{i=1}^{N_{l}} \mathbf{P}{(i)}^{2}$ | | 16. Variance | | $\frac{1}{N}\sum_{i=1}^{N} \left( \mathbf{X}\left( i \right)-\bar{X} \right)^{2}$ |
| **Shape- and Size-based features**  *V*, tumor volume; *A,* surface area of the volume | | | | | |
| 17. Compactness 1 | $\frac{V}{\sqrt{\pi}A^{3/2}}$ | | 18. Compactness 2 | | $36\pi\frac{V^{2}}{A^{3}}$ |
| 19. Maximum 3D diameter | The largest pairwise Euclidean distance between voxels on the surface of the tumor volume. | | 20. Spherical disproportion | | $\frac{A}{4\pi R^{2}}$ |
| 21. Sphericity | $\frac{\pi^{\frac{1}{3}}{(6V)}^{\frac{2}{3}}}{A}$ | | 22. Surface area | | $A=\sum_{i=1}^{Ns} \frac{1}{2}\left\vert a_{i}b_{i}\times a_{i}c_{i} \right\vert$  *Ns*, total number of triangles covering the surface; *a*, *b,* and *c*, triangle vertices |
| 23. Surface to volume ratio | $\frac{A}{V}$ | | 24. Volume | | Number of pixels in the tumor region multiplied by the voxel size |
| **Textural features (gray-level co-occurrence matrix based features)**  $\mathbf{P}(\delta,\alpha)$, co-occurrence matrix for an arbitrary distance δ and direction α; *N_g_* , number of discrete intensity levels in the image; *p_x_*(i), marginal row probabilities; *p_y_*(i), marginal column probabilities; *μ_x_*, mean of *p_x_*; *μ_y_*, mean of *p_y_*; *σ_x_*, standard deviation of *p_x_*; *σ_y_*, standard deviation of *p_y_*; *HXY*, entropy of **P**; *HX*, entropy of *p_x_*; *HY* , entropy of *p_y_*;  $p_{x+y}\left( k \right)=\sum_{i=1}^{N_{g}} \sum_{j=1}^{N_{g}} \mathbf{P}(i,j), i+j=k, k=2,3,\ldots,2N_{g}$;  $p_{x-y}\left( k \right)=\sum_{i=1}^{N_{g}} \sum_{j=1}^{N_{g}} \mathbf{P}(i,j), \left\vert i-j \right\vert=k, k=0,1,\ldots,N_{g}-1$;  $HX=-\sum_{i=1}^{N_{g}} p_{x}\left( i \right)\log_{2} \left( p_{x}\left( i \right) \right),$ $HY=-\sum_{i=1}^{N_{g}} p_{y}\left( i \right)\log_{2} \left( p_{y}\left( i \right) \right)$;  $HXY1=-\sum_{i=1}^{N_{g}} \sum_{j=1}^{N_{g}} \mathbf{P}\left( i,j \right)\log_{2} (p_{x}(i)p_{y}(j))$, $HXY2=-\sum_{i=1}^{N_{g}} \sum_{j=1}^{N_{g}} p_{x}(i)p_{y}(j)\log_{2} (p_{x}(i)p_{y}(j))$ | | | | | |
| 25. Autocorrelation | $\sum_{i=1}^{N_{g}} \sum_{j=1}^{N_{g}} ij\mathbf{P}(i,j)$ | 26. Cluster Prominence | | $\sum_{i=1}^{N_{g}} \sum_{j=1}^{N_{g}} \left[ \boldsymbol{i+j-}\boldsymbol{\mu}_{\boldsymbol{x}}\boldsymbol{-}\boldsymbol{\mu}_{\boldsymbol{y}} \right]^{\boldsymbol{4}}\mathbf{P}(i,j)$ | |
| 27. Cluster Shade | $\sum_{i=1}^{N_{g}} \sum_{j=1}^{N_{g}} \left[ \boldsymbol{i+j-}\boldsymbol{\mu}_{\boldsymbol{x}}\boldsymbol{-}\boldsymbol{\mu}_{\boldsymbol{y}} \right]^{\boldsymbol{3}}\mathbf{P}(i,j)$ | 28. Cluster Tendency | | $\sum_{i=1}^{N_{g}} \sum_{j=1}^{N_{g}} \left[ \boldsymbol{i+j-}\boldsymbol{\mu}_{\boldsymbol{x}}\boldsymbol{-}\boldsymbol{\mu}_{\boldsymbol{y}} \right]^{\boldsymbol{2}}\mathbf{P}(i,j)$ | |
| 29. Contrast | $\sum_{i=1}^{N_{g}} \sum_{j=1}^{N_{g}} \left\vert\boldsymbol{i-j} \right\vert^{\boldsymbol{2}}\mathbf{P}(i,j)$ | 30. Correlation | | $\sum_{i=1}^{N_{g}} \sum_{j=1}^{N_{g}} \frac{ij\mathbf{P}\left( i,j \right)-\mu_{x}(i)\mu_{y}(j)}{\sigma_{x}(i)\sigma_{y}(j)}$ | |
| 31. Difference entropy | $\sum_{i=0}^{N_{g}-1} p_{x-y}(i){log}_{2}\left[ p_{x-y}(i) \right]$ | 32. Dissimilarity | | $\sum_{i=1}^{N_{g}} \sum_{j=1}^{N_{g}} \left\vert\boldsymbol{i-j} \right\vert\mathbf{P}(i,j)$ | |
| 33. Energy | $\sum_{i=1}^{N_{g}} \sum_{j=1}^{N_{g}} \left[ \mathbf{P}(i,j) \right]^{\boldsymbol{2}}$ | 34. Entropy (*HXY*) | | $-\sum_{i=1}^{N_{g}} \sum_{j=1}^{N_{g}} \mathbf{P}\left( i,j \right)\log_{2}(\mathbf{P}\left( i,j \right))$ | |
| 35. Homogeneity 1 | $\sum_{i=1}^{N_{g}} \sum_{j=1}^{N_{g}} \frac{\mathbf{P}\left( i,j \right)}{1+\left\vert i-j \right\vert}$ | 36. Homogeneity 2 | | $\sum_{i=1}^{N_{g}} \sum_{j=1}^{N_{g}} \frac{\mathbf{P}\left( i,j \right)}{1+\left\vert i-j \right\vert^{2}}$ | |
| 37. Informational measure of correlation 1 | $\frac{HXY-HXY1}{max(HX,HY)}$ | 38. Informational measure of correlation 2 | | $\sqrt{1-e^{-2\left( HXY2-HXY \right)}}$ | |
| 39. Inverse Difference Moment Normalized | $\sum_{i=1}^{N_{g}} \sum_{j=1}^{N_{g}} \frac{\mathbf{P}\left( i,j \right)}{1+\left( \frac{\left\vert i-j \right\vert^{2}}{N^{2}} \right)}$ | 40. Inverse Difference Normalized | | $\sum_{i=1}^{N_{g}} \sum_{j=1}^{N_{g}} \frac{\mathbf{P}\left( i,j \right)}{1+\left( \frac{\left\vert i-j \right\vert}{N} \right)}$ | |
| 41. Inverse variance | $\sum_{i=1}^{N_{g}} \sum_{j=1}^{N_{g}} \frac{\mathbf{P}\left( i,j \right)}{\left\vert i-j \right\vert^{2}}, i\neq j$ | 42. Maximum Probability | | $\max\left( \mathbf{P}\left( i,j \right) \right)$ | |
| 43. Sum average | $\sum_{i=2}^{2N_{g}} \left[ i\mathbf{P}_{\boldsymbol{x+y}}(i) \right]$ | 44. Sum entropy | | $-\sum_{i=2}^{2N_{g}} \mathbf{P}_{\boldsymbol{x+y}}(i){log}_{2}\left[ \mathbf{P}_{\boldsymbol{x+y}}(i) \right]$ | |
| 45. Variance | $\sum_{i=1}^{N_{g}} \sum_{j=1}^{N_{g}} \left( i-\mu\right)^{2}\mathbf{P(i,j)}$ |  | |  | |
| **Textural features (gray-level run-length matrix based features)**  $p(i,j\vert\theta)$, (*i,j*)th entry in the given run-length matrix *p* for a direction *θ*; *N_g_*, number of discrete intensity levels in the image; *N_r_*, number of different run lengths | | | | | |
| 46. Short Run Emphasis | $\frac{\sum_{i=1}^{N_{g}} \sum_{j=1}^{N_{r}} \left[ \frac{p\left( i,j\vert\theta\right)}{j^{2}} \right]}{\sum_{i=1}^{N_{g}} \sum_{j=1}^{N_{r}} p\left( i,j\vert\theta\right)}$ | 47. Long Run Emphasis | | $\frac{\sum_{i=1}^{N_{g}} \sum_{j=1}^{N_{r}} j^{2}p\left( i,j\vert\theta\right)}{\sum_{i=1}^{N_{g}} \sum_{j=1}^{N_{r}} p\left( i,j\vert\theta\right)}$ | |
| 48. Gray Level Non-Uniformity | $\frac{\sum_{i=1}^{N_{g}} \left[ \sum_{j=1}^{N_{r}} p\left( i,j\vert\theta\right) \right]^{2}}{\sum_{i=1}^{N_{g}} \sum_{j=1}^{N_{r}} p\left( i,j\vert\theta\right)}$ | 49. Run Length Non-Uniformity | | $\frac{\sum_{j=1}^{N_{r}} \left[ \sum_{i=1}^{N_{g}} p\left( i,j\vert\theta\right) \right]^{2}}{\sum_{i=1}^{N_{g}} \sum_{j=1}^{N_{r}} p\left( i,j\vert\theta\right)}$ | |
| 50. Run Percentage | $\sum_{i=1}^{N_{g}} \sum_{j=1}^{N_{r}} \frac{p\left( i,j\vert\theta\right)}{N_{p}}$ | 51. Low Gray Level Run Emphasis | | $\frac{\sum_{i=1}^{N_{g}} \sum_{j=1}^{N_{r}} \left[ \frac{p\left( i,j\vert\theta\right)}{i^{2}} \right]}{\sum_{i=1}^{N_{g}} \sum_{j=1}^{N_{r}} p\left( i,j\vert\theta\right)}$ | |
| 52. High Gray Level Run Emphasis | $\frac{\sum_{i=1}^{N_{g}} \sum_{j=1}^{N_{r}} i^{2}p\left( i,j\vert\theta\right)}{\sum_{i=1}^{N_{g}} \sum_{j=1}^{N_{r}} p\left( i,j\vert\theta\right)}$ | 53. Short Run Low Gray Level Emphasis | | $\frac{\sum_{i=1}^{N_{g}} \sum_{j=1}^{N_{r}} \left[ \frac{p\left( i,j\vert\theta\right)}{i^{2}j^{2}} \right]}{\sum_{i=1}^{N_{g}} \sum_{j=1}^{N_{r}} p\left( i,j\vert\theta\right)}$ | |
| 54. Short Run High Gray Level Emphasis | $\frac{\sum_{i=1}^{N_{g}} \sum_{j=1}^{N_{r}} \left[ \frac{p\left( i,j\vert\theta\right)i^{2}}{j^{2}} \right]}{\sum_{i=1}^{N_{g}} \sum_{j=1}^{N_{r}} p\left( i,j\vert\theta\right)}$ | 55. Long Run Low Gray Level Emphasis | | $\frac{\sum_{i=1}^{N_{g}} \sum_{j=1}^{N_{r}} \left[ \frac{p\left( i,j\vert\theta\right)j^{2}}{i^{2}} \right]}{\sum_{i=1}^{N_{g}} \sum_{j=1}^{N_{r}} p\left( i,j\vert\theta\right)}$ | |
| 56. Long Run High Gray Level Emphasis | $\frac{\sum_{i=1}^{N_{g}} \sum_{j=1}^{N_{r}} i^{2}j^{2}p\left( i,j\vert\theta\right)}{\sum_{i=1}^{N_{g}} \sum_{j=1}^{N_{r}} p\left( i,j\vert\theta\right)}$ |  | |  | |
| **Textural features (local binary pattern based features)**  **X** denotes the vector of local binary pattern with *N* voxels in the tumor ROIs. The local binary pattern was estimated based on the relations of center pixel with 8 neighbors; $\bar{X},$ the mean of **X**; **P**, the first-order histogram with *N_l_* discrete intensity levels. Equations #1 to 16 (first order statistics) were then applied to yield 16 local binary pattern based features. | | | | | |

**Table S2.** Grid search results of DeepSurv hyper-parameters

| Number of hidden layer | | |
| --- | --- | --- |
| Value | Average C-index | Average time cost (second) |
| 2 | 0.63 | 382 |
| 3 | 0.64 | 455 |
| 4 (Selected) | 0.66 | 435 |
| 5 | 0.66 | 448 |
| 6 | 0.65 | 496 |
| Number of nodes in each hidden layer | | |
| Value | Average C-index | Average time cost (second) |
| 4 | 0.59 | 307 |
| 8 | 0.61 | 384 |
| 16 | 0.67 | 368 |
| 32 (Selected) | 0.68 | 335 |
| 64 | 0.68 | 398 |
| Initial learning rate | | |
| Value | Average C-index | Average time cost (second) |
| 0.001 | 0.62 | 358 |
| 0.005 | 0.65 | 398 |
| 0.01 (Selected) | 0.67 | 385 |
| 0.05 | 0.67 | 392 |
| 0.1 | 0.66 | 453 |
| Learning rate decay | | |
| Value | Average C-index | Average time cost (second) |
| 0.01 (Selected) | 0.65 | 230 |
| 0.001 | 0.65 | 488 |
| 0.0001 | 0.65 | 638 |
| Dropout rate | | |
| Value | Average C-index | Average time cost (second) |
| 0.1 | 0.62 | 457 |
| 0.2 | 0.64 | 423 |
| 0.4 (Selected) | 0.65 | 428 |

The hyper-parameters, including the momentum of 0.9, the epoch of 1000, the optimizer of Adam and the regularization of L2, are fixed.

**Table S3.** Comparisons of clinical characteristics between training and test sets

| **Characteristics** | **Training set** | **Test set** | **p-value** |
| --- | --- | --- | --- |
| **Age** | 67(60-75) | 66(58.5-78) | 0.69 |
| **Gender** |  |  | 0.37 |
| Female, N(%) | 84(44.2) | 74(92.5) |  |
| **Smoking status** |  |  | 0.25 |
| Smoker, N(%) | 54(28.4) | 15(18.8) |  |
| **ECOG PS score** |  |  | 0.22 |
| 0, N(%) | 68(35.8) | 30(37.5) |  |
| 1, N(%) | 102(53.7) | 37(46.2) |  |
| 2, N(%) | 11(5.8) | 11(13.8) |  |
| >2, N(%) | 9(4.7) | 2(2.5) |  |
| **Histology of NSCLC** |  |  | 0.05 |
| Adenocarcinoma, N(%) | 188(90.0) | 75(93.7) |  |
| Squamous cell carcinoma, N(%) | 1 (0.5) | 3(3.8) |  |
| Others, N(%) | 1 (0.5) | 2(2.5) |  |
| **Clinical T stage** |  |  | 0.58 |
| 1, N(%) | 25(13.2) | 10(12.5) |  |
| 2, N(%) | 50(26.3) | 28(35.0) |  |
| 3, N(%) | 29(15.3) | 15(18.8) |  |
| 4, N(%) | 81(42.6) | 25(31.2) |  |
| Not available, N(%) | 5(2.6) | 2(2.5) |  |
| **Clinical N stage** |  |  | 0.92 |
| 0, N(%) | 47(24.8) | 22(27.5) |  |
| 1, N(%) | 13(6.8) | 7(8.8) |  |
| 2, N(%) | 53(28.0) | 22(27.5) |  |
| 3, N(%) | 75(39.7) | 29(36.2) |  |
| Not available, N(%) | 2(0.7) | 0(0) |  |
| **Clinical M stage** |  |  | 0.92 |
| 0, N(%) | 6(3.2) | 4(5.0) |  |
| 1a, N(%) | 60(31.6) | 22(27.5) |  |
| 1b, N(%) | 28(14.7) | 13(16.3) |  |
| 1c, N(%) | 96(50.5) | 41(51.2) |  |
| **Clinical stage** |  |  | 0.96 |
| Stage III, N(%) | 15(7.9) | 8(10.0) |  |
| Stage IVA, N(%) | 78(41.1) | 32(40.0) |  |
| Stage IVB, N(%) | 97(51.0) | 40(50.0) |  |
| **EGFR mutation status** |  |  | 1.00 |
| Exon 19 deletion, N(%) | 83(43.7) | 34(42.5) |  |
| Exon 21 L858R substitution, N(%) | 93(48.9) | 40(50.0) |  |
| Others, N(%) | 14(7.4) | 6(7.5) |  |
| **TKI** |  |  | 0.71 |
| Gefitinib, N(%) | 30(15.8) | 16(20.0) |  |
| Erlotinib, N(%) | 58(30.5) | 27(33.8) |  |
| Afatinib, N(%) | 102 (53.7) | 37(46.2) |  |
| **Adverse drug reaction to EGFR-TKI** |  |  | 0.80 |
| Yes, N(%) | 89(46.8) | 41(51.3) |  |
| **Progression free survival, median(months)** | 11.0 (0-73.1) | 12.6 (0-39.4) | 0.96 |
| **Total protein** |  |  | 0.90 |
| High, N(%) | 59(31.1) | 28(35.0) |  |
| Low, N(%) | 5(2.6) | 1(1.3) |  |
| Not available, N(%) | 126(66.3) | 51(63.7) |  |
| **Mean corpuscular volume** |  |  | 0.85 |
| High, N(%) | 59(31.1) | 26(32.5) |  |
| Normal, N(%) | 55(28.9) | 26(32.5) |  |
| Low, N(%) | 3(1.6) | 2(2.5) |  |
| Not available, N(%) | 73(38.4) | 26(32.5) |  |
| **White blood cells count** |  |  | 0.76 |
| High, N(%) | 54 40(21.1) | 14(17.5) |  |
| Normal, N(%) | 126(66.3) | 59(73.7) |  |
| Low, N(%) | 6(3.2) | 1(1.3) |  |
| Not available, N(%) | 18(9.4) | 6(7.5) |  |

Two sample t-test (for continuous variables) and chi-square test (for categorical variables) are applied to compare the data between the training and test sets.

**Table S4** Characteristics of clinical laboratory test.

| **Characteristics** | **Value** |
| --- | --- |
| **Total protein** |  |
| High, N(%) | 87 (32.2) |
| Low, N(%) | 6 (2.2) |
| Not available, N(%) | 177 (65.6) |
| **Mean corpuscular volume** |  |
| High, N(%) | 85 (31.5) |
| Normal, N(%) | 81 (30.0) |
| Low, N(%) | 5 (1.9) |
| Not available, N(%) | 99 (37.6) |
| **White blood cells count** |  |
| High, N(%) | 54 (20.0) |
| Normal, N(%) | 185 (68.5) |
| Low, N(%) | 7 (2.6) |
| Not available, N(%) | 24 (8.9) |
| **Hemoglobin** |  |
| High, N(%) | 46 (17.0) |
| Normal, N(%) | 202 (74.8) |
| Low, N(%) | 1 (0.4) |
| Not available, N(%) | 21 (7.8) |
| **Platelets count** |  |
| High, N(%) | 38 (14.1) |
| Normal, N(%) | 196 (72.5) |
| Low, N(%) | 15 (5.6) |
| Not available, N(%) | 21 (7.8) |
| **Albumin** |  |
| High, N(%) | 107 (39.6) |
| Normal, N(%) | 34 (12.6) |
| Not available, N(%) | 129 (47.8) |
| **Lactic dehydrogenase** |  |
| High, N(%) | 53 (19.6) |
| Normal, N(%) | 46 (17.1) |
| Not available, N(%) | 171 (63.3) |
| **C-Reactive protein** |  |
| High, N(%) | 71 (26.3) |
| Normal, N(%) | 26 (9.6) |
| Not available, N(%) | 173 (64.1) |
| **Creatinine** |  |
| High, N(%) | 34 (12.6) |
| Normal, N(%) | 211 (78.1) |
| Not available, N(%) | 25 (9.3) |
| **Blood urea nitrogen** |  |
| High, N(%) | 42 (15.6) |
| Normal, N(%) | 178 (65.9) |
| Not available, N(%) | 50 (18.5) |
| **Total bilirubin** |  |
| High, N(%) | 7 (2.6) |
| Normal, N(%) | 159 (58.9) |
| Not available, N(%) | 104 (38.5) |
| **Alanine aminotransferase** |  |
| High, N(%) | 23 (8.5) |
| Normal, N(%) | 205 (75.9) |
| Not available, N(%) | 42 (15.6) |
| **Aspartate transferase** |  |
| High, N(%) | 15 (5.6) |
| Normal, N(%) | 154 (57.0) |
| Not available, N(%) | 101 (37.4) |

**Table S5.** Identified features for the model training in each DeepSurv model.

| **Feature Name** | **Feature Type** | **Wavelet Filtering** |
| --- | --- | --- |
| **Clinical Model** | | |
| Stage N | Clinical | - |
| Stage M | Clinical | - |
| Histology | Clinical | - |
| Total protein | Clinical | - |
| Mean Corpuscular Volume | Clinical | - |
| **Radiomic Model** | | |
| Compactness | Geometric | - |
| Inverse Difference Moment Normalized | GLCM | LHL |
| Inverse Difference Moment Normalized | GLCM | HLL |
| Inverse Difference Moment Normalized | GLCM | HHL |
| Inverse Difference Moment Normalized | GLCM | HHH |
| **Combined Model** | | |
| Stage N | Clinical | - |
| Stage M | Clinical | - |
| Histology | Clinical | - |
| Total Protein | Clinical | - |
| Mean Corpuscular Volume | Clinical | - |
| Compactness | Geometric | - |
| Inverse Difference Moment Normalized | GLCM | LHL |
| Inverse Difference Moment Normalized | GLCM | HLL |
| Inverse Difference Moment Normalized | GLCM | HHL |
| Inverse Difference Moment Normalized | GLCM | HHH |

**GLCM**: gray-level co-occurrence matrix. In the column of wavelet filtering, L represents a low-pass filter, and H represents a high-pass filter. The combination of L and H letters stands for the filter type applied to the three image axes in order.


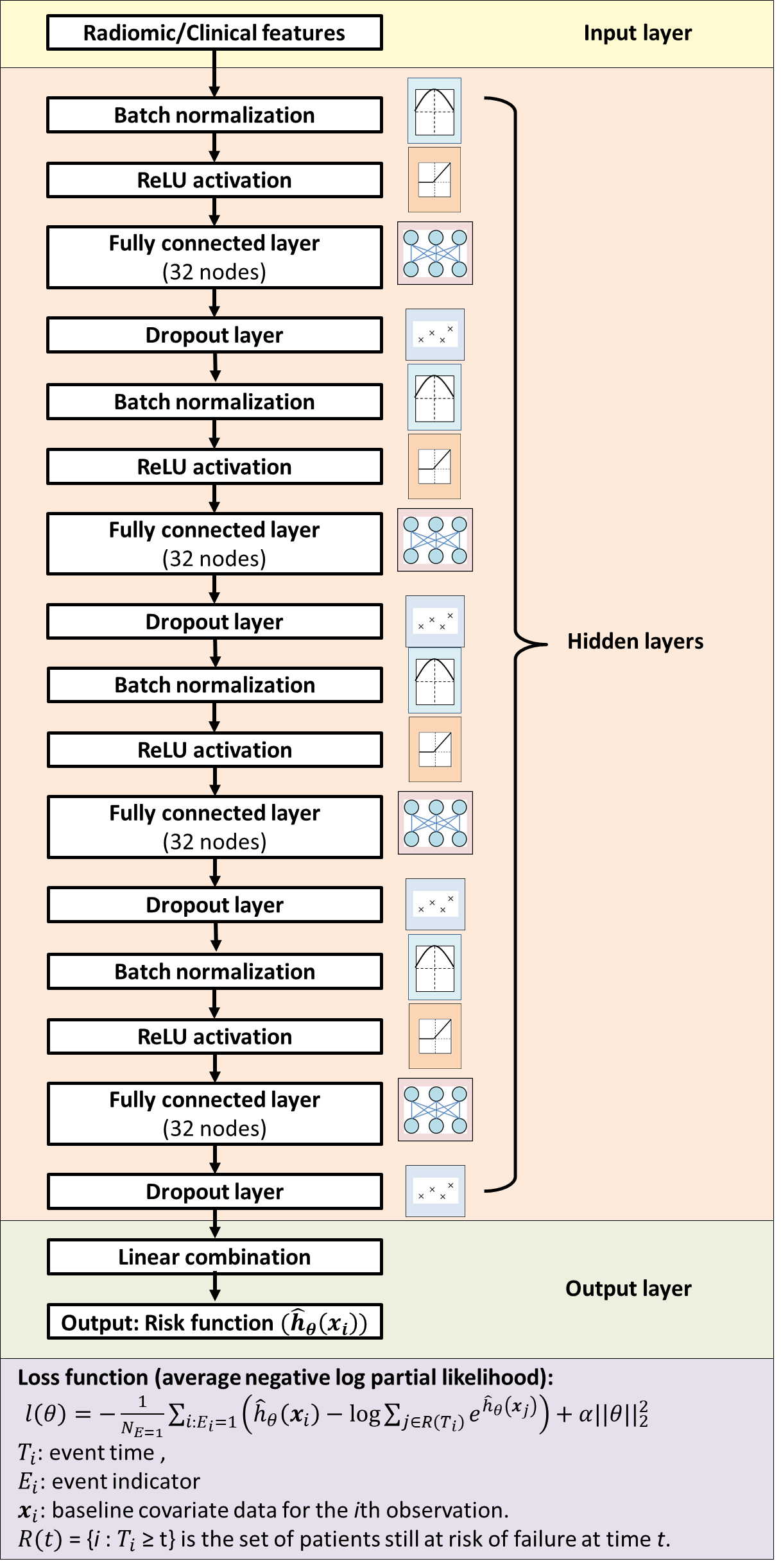


**Figure S1.** **The architecture of applied DeepSurv model.** The applied DeepSurv models using radiomic and clinical features as inputs. The network propagates the inputs through hidden layers with specified weights. Each fully connected layer contains 32 nodes followed by 40% of dropout. The minimization of the average negative log partial likelihood is the training objective.


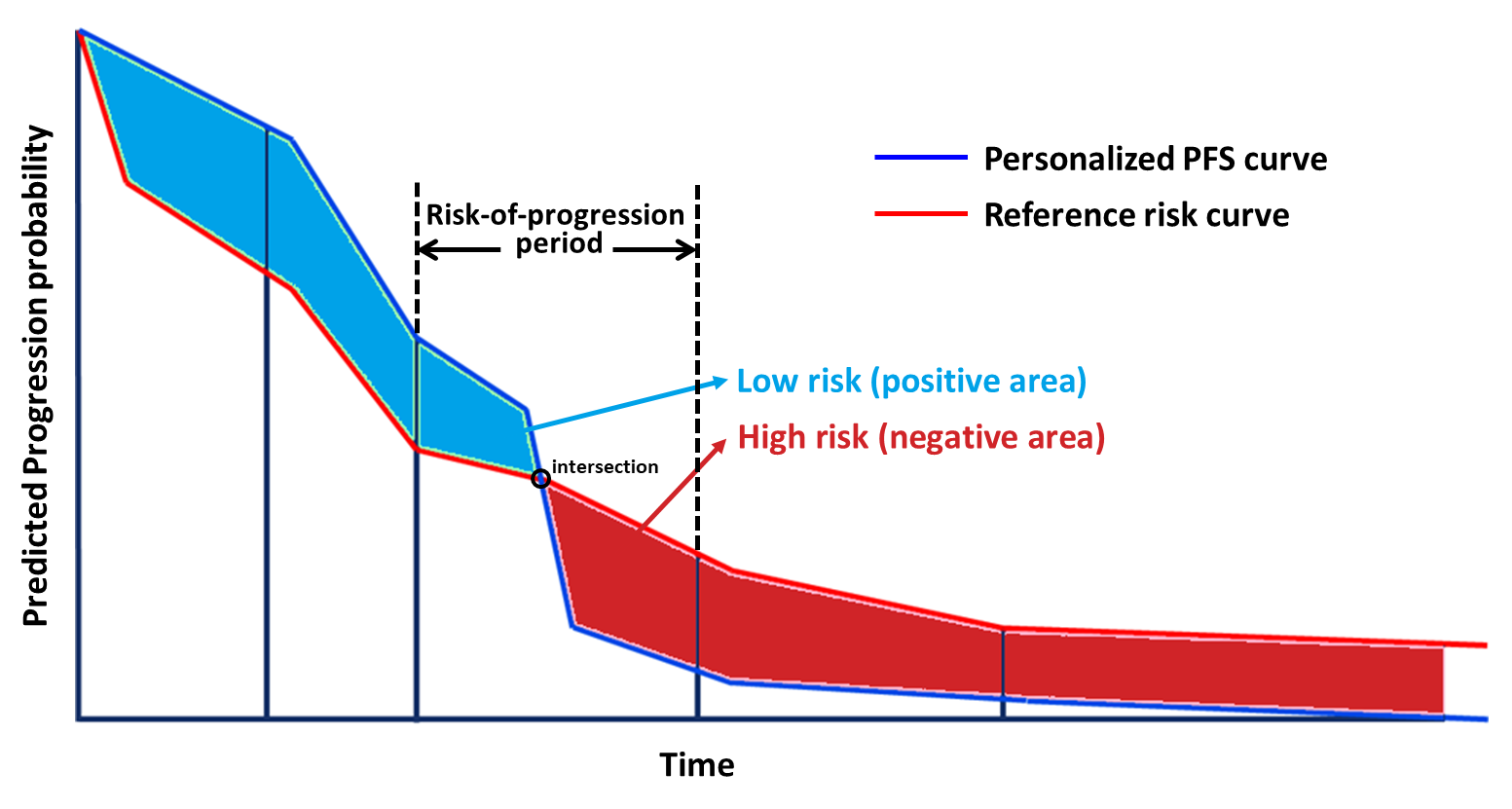


**Figure S2.** **Schematic diagram of predictive risk-of-progression period in DeepSurv model.** Each patient is assigned an estimated personalized PFS curve based on the Cox log-risk function output from the DeepSurv model (blue line). A reference risk curve of the DeepSurv model can be obtained by fitting the thresholds of PFS prediction at each important time point (red line). When a period shows that the area under personalized PFS curve lower than the reference risk curve, that period is estimated as the risk-of-progression period of patient.
